# Intestinal microbial community assessment in patients with different forms of epilepsy and autoimmune encephalitis: An exploratory study

**DOI:** 10.1080/29933935.2026.2679348

**Published:** 2026-05-28

**Authors:** Diana Marcela Mejía-Granados, Tânia Kawasaki de Araújo, Stefanie Malan-Müller, Patrícia Aline Oliveira Ribeiro de Aguiar Araújo, Luciano Henrique Braz dos Santos, Clarissa Lin Yasuda, Marina Koutsodontis Machado Alvim, Benilton de Sá Carvalho, Fernando Cendes, Iscia Lopes-Cendes

**Affiliations:** a Department of Medical Genetics and Genomic Medicine, School of Medical Sciences, University of Campinas (UNICAMP), Campinas, SP, Brazil; b Department of Pharmacology and Toxicology, Faculty of Medicine, University Complutense of Madrid (UCM); Research Institute of Hospital 12 de Octubre (Imas12); Instituto Universitario de Investigación Neuroquímica (IUIN-UCM), Madrid, Spain; c Biomedical Research Network Centre in Mental Health, Institute of Health Carlos III (CIBERSAM, ISCIII), Madrid, Spain; d Brazilian Institute of Neuroscience and Neurotechnology (BRAINN), Campinas, SP, Brazil; e Department of Neurology, School of Medical Sciences, University of Campinas, Campinas, SP, Brazil; f Department of Statistics, Institute of Mathematics, Statistics, and Scientific Computing, University of Campinas (UNICAMP), Campinas, SP, Brazil

**Keywords:** Gut microbiome, 16S rRNA sequencing, epilepsy, autoimmune encephalitis

## Abstract

Despite the availability of antiseizure medications (ASMs), approximately one-third of patients with epilepsy remain refractory to treatment. Evidence suggests that the gut microbiota modulates central nervous system function through neuroimmune and metabolic pathways. We characterized the fecal microbiome of patients with epilepsy and autoimmune encephalitis (AE) using 16S rRNA sequencing. Ninety-six participants from a tertiary hospital in Brazil were included: mesial temporal lobe epilepsy (MTLE; *n* = 38), genetic generalized epilepsy (GGE; *n* = 11), AE (*n* = 10), and healthy controls (*n* = 37). Microbial community composition differed between treatment-responsive and refractory patients. Compared with responsive individuals (*n* = 16), refractory patients (*n* = 43) showed lower relative abundances of *Agathobaculum*, *Bacteroides*, *Bilophila*, and *Coprobacter*, and higher abundance of *Guopingia*. Dietary fiber intake was negatively associated with *Enterocloster* and *Frisingicoccus*. Riboflavin (vitamin B2) and niacin (vitamin B3) were positively associated with *Schaalia* and *Hominimerdicola*, respectively. Functional predictions indicated that valproic acid use and treatment responsiveness were associated with increased microbial potential for GABA synthesis and tryptophan degradation, and reduced potential for dopamine and histamine degradation. These findings link the gut microbiome to epilepsy subtype and treatment response.

## 1. Introduction

The International League Against Epilepsy (ILAE) defines epilepsy as a group of chronic, heterogeneous, multifactorial disorders characterized by a persistent predisposition to generate spontaneous epileptic seizures.^1^ Epilepsy affects approximately 70 million people worldwide and ranks among the most prevalent neurological conditions.^2^ In addition to its neurological manifestations, epilepsy encompasses neurobiological, cognitive, and psychosocial dimensions, with consequences that extend well beyond its substantial economic and public health burden.^1^ ^,^ ^3^

Epilepsy is a multifactorial disease with a substantial genetic predisposition.^4^ Despite its steady global prevalence during the last two decades, over 75% of individuals with uncontrolled epilepsy reside in middle and low-income countries. The significant treatment gap in these developing nations continues to contribute to heightened disability rates. This represents a serious issue, not only from a public health perspective but also in terms of quality of life, morbidity, and mortality.^5^

Diagnosing epilepsy in clinical practice remains challenging and carries significant implications, given the absence of a gold standard and the broad phenotypic spectrum. When feasible, clinical characterization should be conducted across three levels: (i) seizure type, (ii) epilepsy type, and (iii) potential syndromic classification. This approach enables a more dynamic and precise diagnosis.^3^ ^,^ ^6^ Advances in high-resolution neuroimaging, molecular diagnostics, and genetic testing have refined the understanding of epilepsy etiology, which now includes structural, metabolic, genetic, infectious, immune, and neurodegenerative causes. Nevertheless, the underlying cause remains unidentified in approximately 36% of cases.^2^ ^,^ ^6^ ^,^ ^7^

The gut–brain axis is a bidirectional communication network linking the central nervous system (CNS) and the enteric nervous system (ENS), mediated in part by the gut microbiota. This axis has been implicated in various neurological disorders, including epilepsy.^8^ ^,^ ^9^ Rodent models and clinical studies involving dietary interventions, particularly the ketogenic diet, have highlighted the potential role of intestinal microorganisms in modulating seizure activity.^10^ ^,^ ^11^ Notably, individuals with epilepsy often exhibit gut microbiota imbalances compared to healthy controls, which may influence brain function and contribute to seizure susceptibility.^12^ ^,^ ^13^

Given that different epilepsy subtypes may present distinct clinical profiles and microbiome interactions, this study aimed to characterize the intestinal microbiota composition in patients with three forms of epilepsy: (i) mesial temporal lobe epilepsy (MTLE), the most common form of focal epilepsy; (ii) genetic generalized epilepsy (GGE), which is believed to have a strong genetic basis; and (iii) autoimmune encephalitis (AE), in which neuronal antibodies target brain proteins, leading to inflammation and seizures. These profiles were compared with those of healthy controls. Metagenomic analysis was conducted using a 16S rRNA marker gene approach, followed by functional predictions to identify microbial metabolic pathways potentially associated with epilepsy and autoimmune encephalitis.

## 2. Results

### 2.1. Demographics and clinical aspects of the cohort

**Figure 1.**
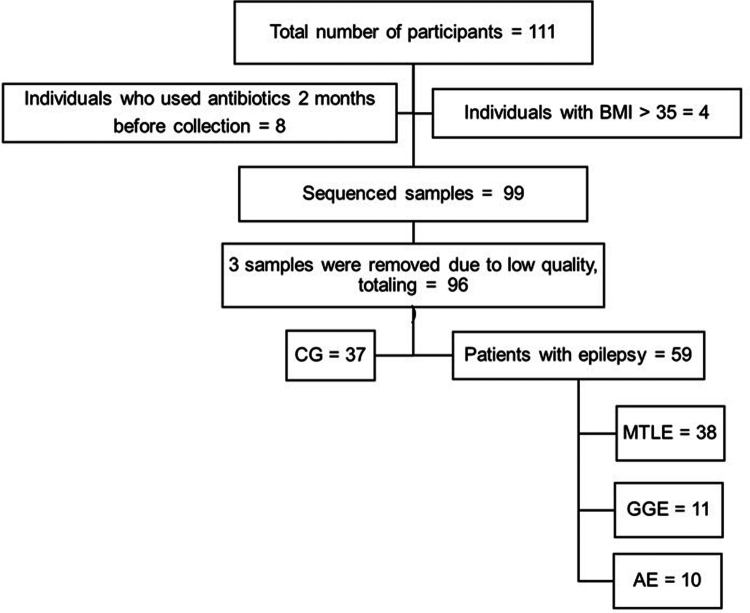
Patient enrollment flowchart illustrating the application of inclusion and exclusion criteria.

A total of 111 samples were collected for this study. After excluding participants who did not meet the inclusion criteria, 99 individuals were included in the analysis and distributed into four groups based on epilepsy etiology: mesial temporal lobe epilepsy (MTLE), genetic generalized epilepsy (GGE), autoimmune encephalitis (AE), and a control group (CG) (Figure 1).

Participants were matched by sex and age with healthy individuals, preferably from the same family unit (e.g., spouses), to minimize variability related to sociodemographic factors and dietary habits. The control group consisted of individuals without known neurological diseases, primarily unrelated family members of the patients.

Inclusion criteria for controls were: (1) age between 18 and 70 y and (2) body mass index (BMI) between 18 and 35, according to World Health Organization criteria. The same exclusion criteria were applied to both patients and controls, including: (1) history of inflammatory bowel disease (Crohn’s disease, ulcerative colitis, irritable bowel syndrome) or malabsorption syndromes; (2) use of high-dose corticosteroids, anti-inflammatory drugs, immunosuppressants, or antibiotics within two months prior to sample collection; (3) high consumption of probiotics; (4) alcohol intake within 24 hours prior to sampling; and (5) history of gastrointestinal surgery.

After filtering the reads, three low-quality samples were removed, belonging to two controls and one from the MTLE group. Clinical characteristics such as sex, age, body mass index (BMI), and Bristol stool scale (BSS) classification were not significantly different between the control (CG) and epilepsy (MTLE + GGE + AE) groups. However, the dietary survey revealed significant differences in the usual intake of micronutrients such as potassium (*p* = 0.010), zinc (*p =* 0.042), vitamin B1 (*p* = 0.012), vitamin B6 (*p* = 0.007) and vitamin E (*p* = 0.007), with a higher intake in the CG group than in the combined patient groups (MTLE + GGE + AE) (Table 1. Supplemental material).

Similarly, higher use of antiseizure medications (ASMs) was observed in patients with MTLE than in those with other forms of epilepsy (*p* = 0.028). When comparing each class of ASM between the subgroups of patients (MTLE + GGE + AE), we observed higher clobazam (CLB) (*p* < 0.001), carbamazepine (CBZ) (*p* = 0.039), and lamotrigine (LTG) (*p* = 0.041) use in the MTLE subgroup. Moreover, the refractory phenotype (*p* < 0.001) (Fisher’s test) and hippocampal atrophy (HA) (predominantly located in the left temporal lobe) were strongly associated with the MTLE subgroup (*p* < 0.001).

The three epilepsy subgroups (MTLE + GGE + AE) were well matched. After comparison, no significant differences in the rates of brain surgery or common psychiatric comorbidities, such as major depression or anxiety, were found (Table 2. Supplemental material).

### 2.2. Alpha diversity is preserved across groups, while gut microbial composition differs between treatment-responsive and refractory epilepsy

We applied a Kruskal-Wallis test to assess differences in Simpson's diversity across all four groups simultaneously (MTLE, AE, GGE, and Control). The Kruskal-Wallis test revealed no statistically significant difference in Simpson's diversity across the four classification groups (χ² = 5.99, df = 3, *p* = 0.112).

**Figure 2.**
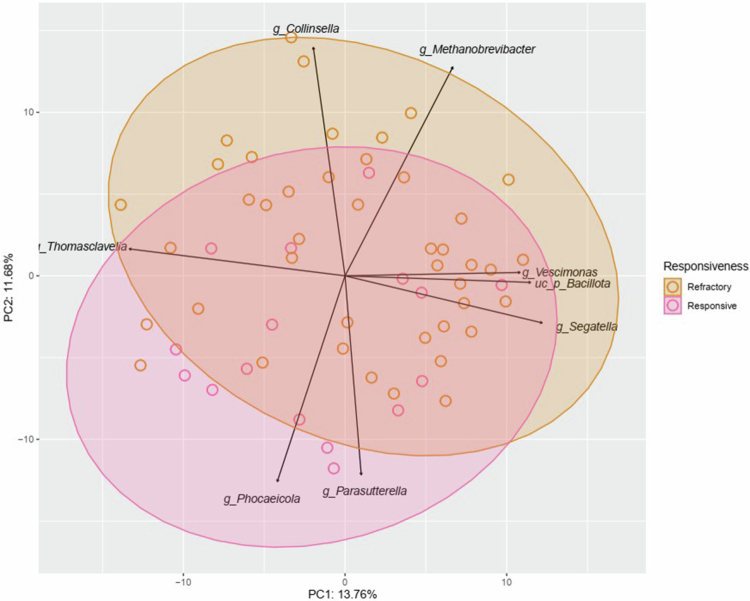
Community variation was visualized using principal component analysis (PCA) of genus-level Aitchison distances (Euclidean distance on clr-transformed data) from 43 treatment-resistant refractory patients (Refractory; orange) and 16 patients who responded to treatment (Responsive; pink). Arrows represent the taxa contributing most to the variation in beta-diversity, with each arrow pointing in the direction where the corresponding taxon values increased the most across all individuals. PCA axes 1 and 2 explained 13.8% and 11.7% of the variation, respectively. clr, centered log-ratio; g_, genus-level taxa; PC1, principal component analysis axis 1; PC2, principal component analysis axis 2; PCA, principal components analysis.

There were differences in the overall gut microbial composition (beta-diversity) between treatment-responsive epilepsy patients (*n* = 16) and refractory epilepsy patients (*n* = 43) (distance-based redundancy analysis (dbRDA)*, q* = 0.05, *R* ^*2*^ = 0.02, *n* = 59) (Figure 2).

### 2.3. Taxa associated with epilepsy classifications

**Figure 3.**
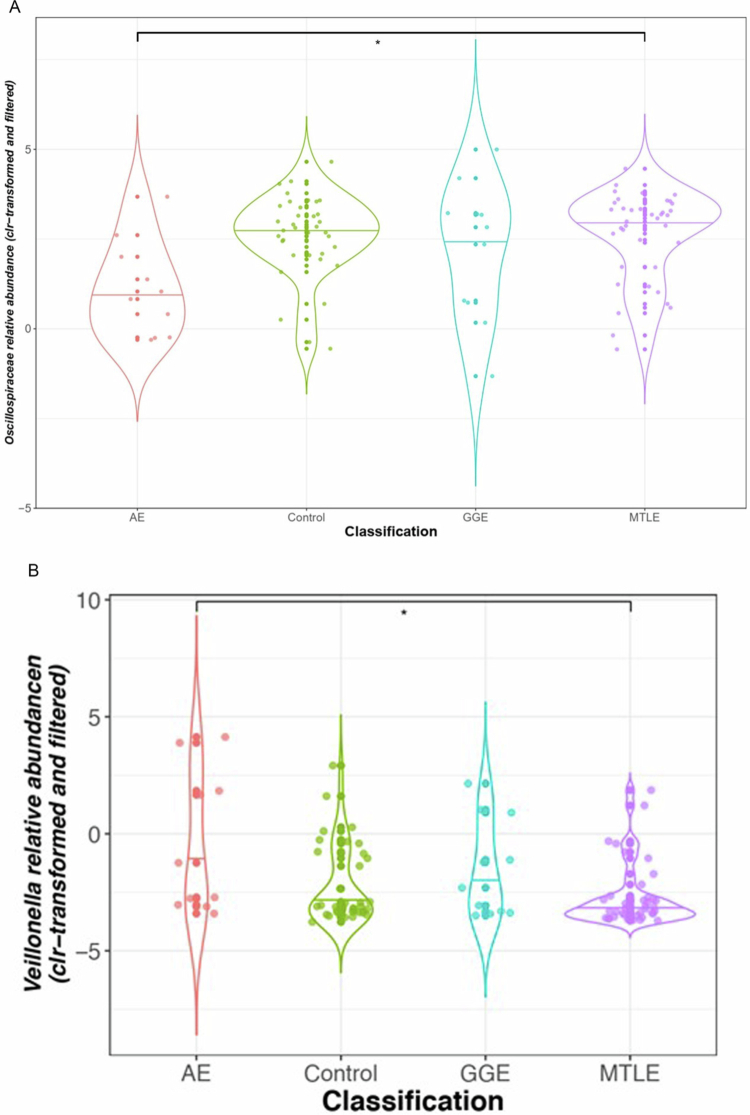
The relative abundance of the Oscillospiraceae family (A) was lower, and that of the *Veillonella* genus (B) was higher in the AE than in the MTLE subgroups. Horizontal lines on the violin plots indicate the median, and the thicker part of the violin around the median represents the interquartile range (IQR). AE - autoimmune encephalitis, MTLE - mesial temporal lobe epilepsy, * *q* = 0.11.

Patients with AE had a lower relative abundance of the family Oscillospiraceae compared to controls (GLM *q* = 0.32, *β* *=* −1.64, *n* = 48) and MTLE (GLM *q* = 0.11, *β* *=* −1.64, *n* = 48) groups; however, only the comparison with the MTLE group came close to statistical significance. A higher relative abundance of *Veillonella* was noted in the AE group compared to the control (GLM *q* = 0.32, *β* *=* 2.32, *n* = 48) and the MTLE groups (GLM *q* = 0.11, *β* *=* 2.32, *n* = 48), although only the MTLE comparison came close to significance (Figure 3A, 3B).

### 2.4. Taxa associated with treatment response

**Figure 4.**
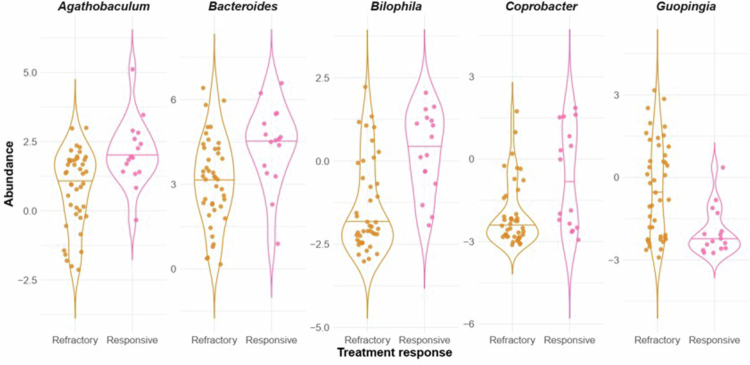
The relative abundances of the genera *Agathobaculum*, *Bacteroides*, *Bilophila*, and *Coprobacter* were lower in patients with refractory epilepsy, compared to responsive patients. In contrast, *Guopingia* demonstrated a higher relative abundance in the same group of patients. Horizontal lines on the violin plots indicate the median, and the thicker part of the violin around the median represents the interquartile range (IQR). Pink: responsive phenotype; orange: refractory phenotype. *q* = 0.1.

Compared with treatment-responsive patients (*n* = 16), refractory patients (*n* = 43) had a significantly lower relative abundance of several genera, including *Agathobaculum* (GLM *q* = 0.1, *β* *=* −1.34, *n* = 59), *Bacteroides* (GLM *q* = 0.1, *β* *=* −1.52, *n* = 59), *Bilophila* (GLM *q* = 0.1, *β* *=* −1.56, *n* = 59), and *Coprobacter* (GLM *q* = 0.1, *β* *=* −1.54, *n* = 59), and higher relative abundance of *Guopingia* (GLM *q* = 0.1, *β* *=* 1.62, *n* = 59) (Figure 4).

### 2.5. Associations between microbiome taxonomic abundance and dietary vitamins, fiber, and short-chain fatty acids

**Figure 5.**
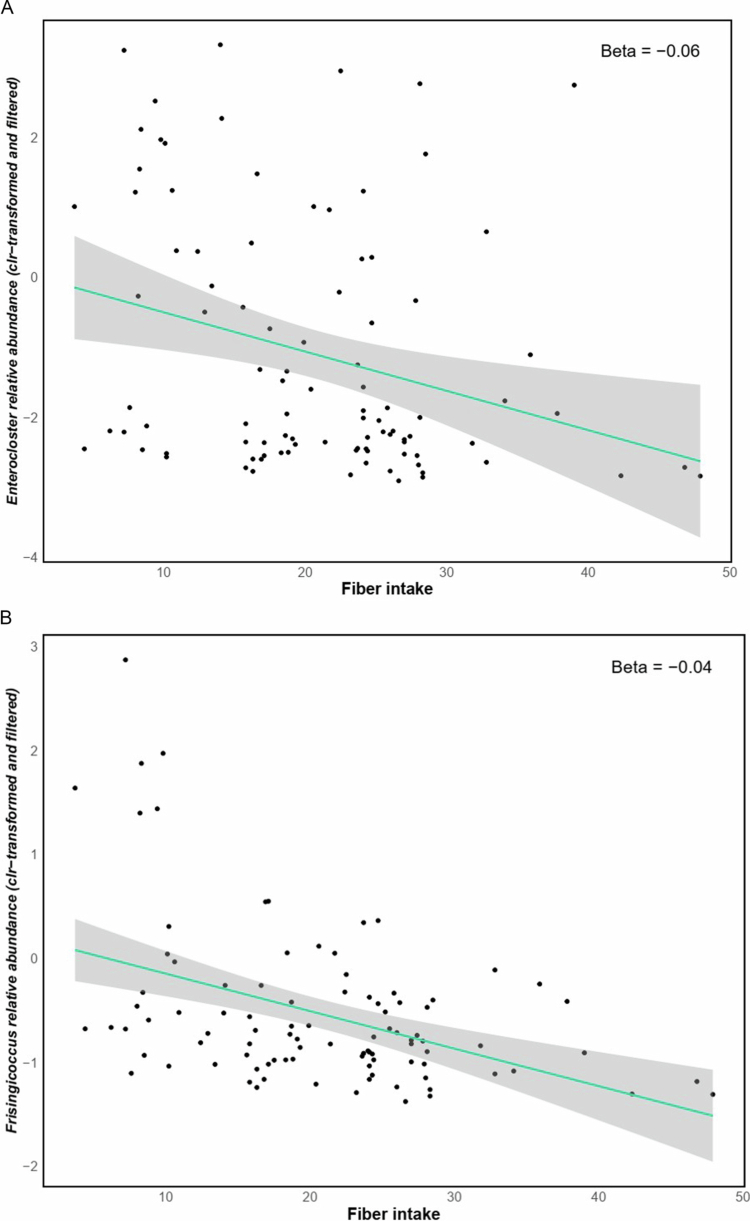
Linear regression analysis showed a negative association between fiber intake (grams of fiber consumed per day) and the relative abundances of *Enterocloster* (A) and *Frisingicoccus* (B).

Fiber intake was negatively associated with the relative abundance of *Enterocloster* (GLM *q* = 0.1, *β* *=* −0.06, *n* = 96) and *Frisingicoccus* (GLM *q* = 0.002, *β* *=* −0.04, *n* = 96) genera (Figure 5A and 5B). In addition, the estimated levels of SCFAs (based on dietary fiber intake data) were positively associated with the *Adlercreutzia* genus (GLM *q* = 0.081, *β* *=* 0.071, *n* = 96) (Figure 6A). Furthermore, daily vitamin intake (estimated from dietary data using WebDiet software) was associated with taxonomic abundance; Riboflavin (vitamin B2) was positively associated with *Schaalia* (GLM *q* = 0.06, *β* *=* 0.66, *n* = 96) (Figure 6B) and Niacin (vitamin B3), was positively associated with *Hominimerdicola* abundance (GLM *q* = 0.09, *β* *=* 0.03, *n* = 96) (Figure 6C).

Figure 6.Linear regression analysis indicated positive associations between dietary SCFAs and *Adlercreutzia* genus (A). Moreover, dietary-derived estimations of riboflavin (vitamin B2) (mg/day) and niacin (vitamin B3) (mg/day) were positively associated with the *Schaalia* (B) and *Hominimerdicola* (C) genera, respectively.Scatter plots showing correlations: A) Short chain fatty acid levels vs. abundance; B) Vitamin B2 intake vs. abundance; C) Vitamin B3 intake and Hominimericola relative abundance. Trendlines with shaded confidence intervals are included.

**Figure:**
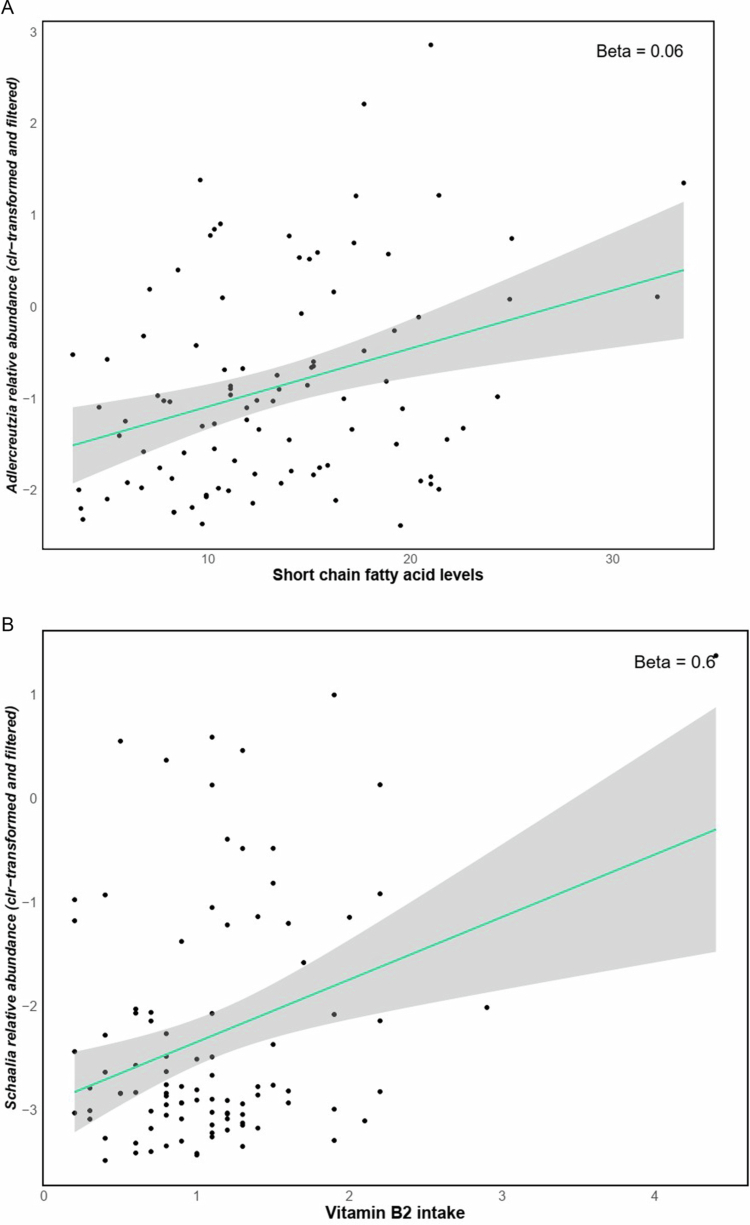

**Figure:**
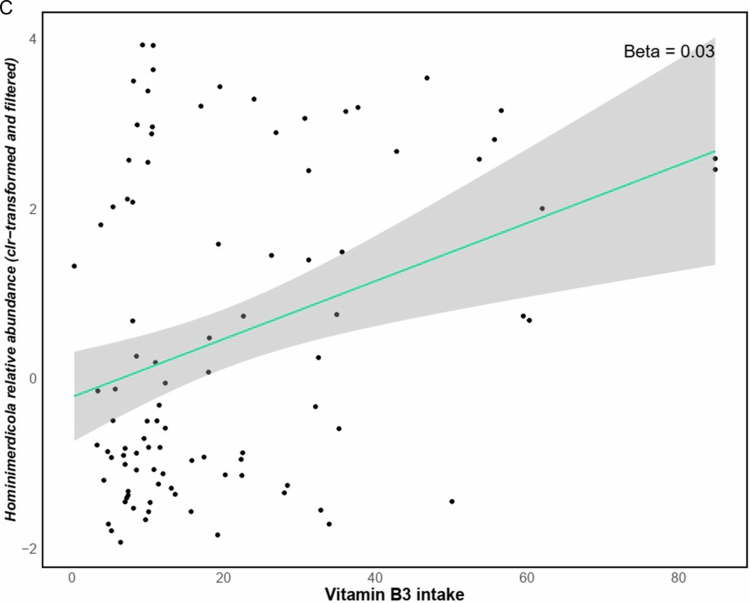

### 2.6. Functional potential of the gut microbiota on metabolism and response to ASMs

Functional predictions of gut-brain modules (GBMs) revealed significant associations with specific microbial functions in patients using VPA, including higher tryptophan degradation (GLM *q* = 0.08, *β* *=* 0.52, *n* = 59), propionate synthesis (GLM *q* = 0.06, *β* *=* 0.72, *n* = 59), GABA synthesis (GLM *q* = 0.06, *β* *=* 0.77, *n* = 59), acetate degradation (GLM *q* = 0.1, *β* *=* 0.53, *n* = 59), and menaquinone synthesis (vitamin K2) (GLM *q* = 0.06, *β* *=* 0.67, *n* = 59).

**Figure 7.**
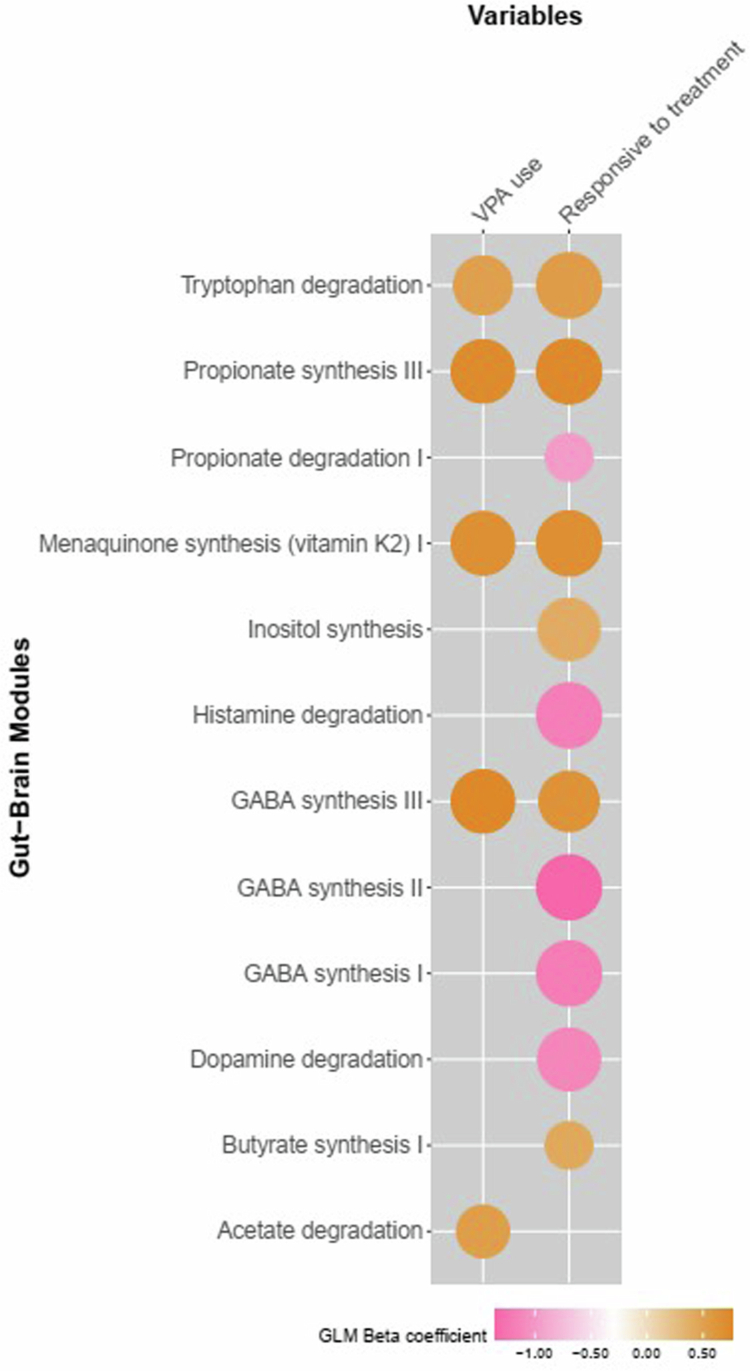
Summary graphic highlighting common and unique GBMs associated with VPA use and treatment response in the patient cohort (*n* = 59). Positive associations are shown in shades of orange, negative associations are shown in shades of pink, the color intensity is proportional to the standardized GLM *β* coefficients, and the point size is proportional to the *q*-values (FDR corrected *p*-values). VPA use (*n* = 19, 32%) and treatment-responsive (*n* = 27%). VPA - valproate, GABA - gamma-aminobutyric acid.

Treatment-responsive patients were predicted to have higher functional potential for inositol synthesis (GLM *q* = 0.06, *β* *=* 0.40, *n* = 59), propionate synthesis (GLM *q* = 0.05, *β* *=* 0.73, *n* = 59), butyrate synthesis (GLM *q* = 0.09, *β* *=* 0.43, *n* = 59), tryptophan degradation (GLM *q* = 0.05, *β* *=* 0.54, *n* = 59), GABA synthesis III (via the polyamine degradation pathway) (GLM *q* = 0.07, *β* *=* 0.64, *n* = 59), and menaquinone synthesis (vitamin K2) (GLM *q* = 0.05, *β* *=* 0.67, *n* = 59) and lower functional potential for propionate degradation (GLM *q* = 0.09, *β* *=* −0.96, *n* = 59), histamine degradation (GLM *q* = 0.05, *β* *=* −1.16, *n* = 59), dopamine degradation (GLM *q* = 0.06, *β* *=* −1.10, *n* = 59), GABA synthesis I (via the glutamate decarboxylation pathway) (GLM *q* = 0.05, *β* *=* −1.17, *n* = 59) and GABA synthesis II (via the putrescine degradation pathway) (GLM *q* = 0.05, *β* *=* −1.34, *n* = 59). Figure 7 illustrates the full set of predicted GBMs linked to treatment response and VPA use.

## 3. Discussion

The gut–brain axis has emerged as a relevant pathway in neuropsychiatric disorders; however, its role in epilepsy remains incompletely understood. In this exploratory study, we identified microbiota patterns associated with epilepsy subtypes, treatment response, dietary intake, and predicted microbial function. These findings should be interpreted as hypothesis-generating and aimed at informing future mechanistic studies rather than to establish causal or clinically actionable relationships.

We did not observe significant differences in alpha diversity across MTLE, AE, GGE, and control groups. This is consistent with current evidence suggesting that microbiome alterations in epilepsy are not consistently reflected in global diversity metrics, but rather in compositional changes.^14^ ^,^ ^15^ Variability across studies may be explained by differences in cohort characteristics, antiseizure medication (ASM) exposure,^16^ dietary factors, and methodological approaches, including the choice of diversity indices. In addition, Simpson’s index emphasizes evenness and dominant taxa and may be less sensitive to subtle changes in richness, which could partly explain the absence of significant findings in our dataset.

Data on autoimmune encephalitis (AE) remain limited, particularly in adult populations. AE patients showed visually distinct relative abundances of Oscillospiraceae and *Veillonella* compared to both MTLE and Control groups (Figure 3A, 3B), with MTLE and Control displaying similar distributions. While the pairwise comparison between AE and Control did not reach statistical significance following FDR correction (*q* = 0.32), likely reflecting limited power due to smaller AE group size, the directional trend was consistent with the significant AE vs MTLE finding (q = 0.11), which narrowly missed the statistical significance of q ≤ 0.1.

Oscillospiraceae are butyrate-producing taxa involved in gut barrier integrity and inflammatory regulation.^17^ ^,^ ^18^ Butyrate plays a critical role in maintaining intestinal homeostasis by fueling enterocyte metabolism, suppressing pro-inflammatory cytokine production, and promoting epithelial repair—functions that may be disrupted in autoimmune encephalitis (AE). Loss of butyrate-producing taxa may impair SCFA-mediated signaling, compromising gut barrier integrity and potentially increasing seizure susceptibility.^19^ ^,^ ^20^ *Veillonella* has been associated with mucosal barrier dysfunction and neuroimmune activation.^21^ ^,^ ^22^ This aligns with prior studies in anti-NMDAR encephalitis, which reported altered gut composition and increased *Veillonella* abundance.^21-23^ Since our study was based on 16S rRNA sequencing and did not include metabolomic analyses, shotgun metagenomics, or direct quantification of short-chain fatty acids (SCFAs), this fact limits our ability to infer metabolic activity or inflammatory status.

Beta diversity analysis revealed differences between treatment-responsive and refractory patients, indicating that microbial composition may be associated with treatment response. Refractory epilepsy was characterized by reduced abundance of taxa involved in short-chain fatty acid (SCFA) production and bile acid metabolism, including *Bacteroides*, *Agathobaculum*, *Bilophila*, and *Coprobacter*, and increased abundance of *Guopingia.* ^24-30^ These taxa have been implicated in neuroimmune modulation, neurotransmitter production, and metabolic regulation.^26, 31-39^ Rather than representing isolated taxonomic changes, the observed microbial differences appear to converge on key functional pathways relevant to seizure control. However, these associations remain correlative and do not establish causality.

We observed an association between bilateral hippocampal atrophy and increased abundance of *Holdemania*. This genus, though understudied, is involved in mucin breakdown and SCFA synthesis.^40^ In rodent models, elevated T2 signals in the hippocampus and striatum have been linked to chronic neuroinflammation and blood–brain barrier injury.^23^ These findings suggest that microbial alterations may contribute to structural brain changes in epilepsy.

Fiber intake negatively correlated with *Enterocloster* and *Frisingicoccus*, taxa linked to ethanol and TMAO production, respectively.^41-44^ *Enterocloster* has been associated with non-alcoholic fatty liver disease, type 2 diabetes, and autism. *Frisingicoccus*, linked to high-saturated fat/low -fiber diets, contributes to TMAO synthesis, a metabolite implicated in cardiovascular and renal disease.

Dietary factors also appeared to shape microbial composition. Fiber intake was inversely associated with taxa linked to unfavorable metabolic profiles, while estimated SCFA levels correlated with *Adlercreutzia*, a genus involved in carbohydrate metabolism and microbial stability.^45^ In experimental models, *Adlercreutzia* has been associated with increased microbial diversity and reduced opportunistic pathogens, suggesting potential anti-inflammatory effects^46^. However, as SCFA levels were inferred from dietary data, and in the absence of metabolomics or direct metabolite quantification, no definitive conclusions can be drawn regarding actual biochemical activity.

Vitamin B2 intake correlated with *Schaalia* abundance, a nitrite-producing bacterium contributing to nitric oxide synthesis and mitochondrial health.^47-50^ Riboflavin deficiency has been linked to oxidative stress and intractable epilepsy.^51^ *Schaalia* and *Actinomyces* are involved in riboflavin biosynthesis, supporting redox balance and myelin integrity.

Vitamin B3 intake was associated with *Hominimerdicola*, a genus with limited characterization but potential involvement in energy metabolism. These findings suggest that dietary vitamins may shape microbial communities with neuroprotective potential.

Functional predictions based on gut–brain modules (GBMs) indicated enrichment of pathway activity related to neurotransmitter metabolism and energy homeostasis in treatment-responsive patients and in those receiving valproic acid (VPA). Notably, enrichment of GABA synthesis pathways in VPA users is consistent with the known mechanism of action of this drug, which enhances GABAergic neurotransmission.^52^ ^,^ ^53^ This raises the hypothesis that the microbiome may contribute to or modulate pharmacokinetic effects. Similarly, enrichment of pathways related to SCFA production and menaquinone (vitamin K2) biosynthesis suggests potential links to mitochondrial function and neuronal death and metabolism.^54-56^ It has been suggested that its molecular target likely includes proteins involved in quinone oxidation/reduction within the electron transport chain. Additionally, the mechanism of action for vitamin K analogs probably involves maintaining mitochondrial balance and ATP levels and their capacity to regulate neuronal calcium, which may significantly contribute to their seizure-attenuating effects.^56^ However, these functional inferences are based on 16S rRNA taxonomic data and therefore require confirmation through metabolomics or shotgun metagenomics.

The GABA Synthesis III (shunt pathway) was enriched in both VPA users and treatment-responsive patients, reflecting the mode of action of this class of ASM. By inhibiting GABA transaminase (GABA-T), the enzyme responsible for GABA degradation,^52^ ^,^ ^53^ these patients likely experience elevated GABA levels and reduced GABA breakdown, leading to increase inhibitory neurotransmission and effective seizure control. Interestingly, treatment-responsive patients showed lower enrichment of certain upstream GABA synthesis pathways [GABA Synthesis I (glutamate decarboxylation) and GABA Synthesis II (polyamine degradation)], which may reflect normalization of GABAergic signaling, whereas refractory patients exhibited enrichment of neurotransmitter degradation pathways, suggesting a shift toward excitatory balance.^57^ These findings provide a framework for generating hypotheses regarding microbiome-related mechanisms of treatment response, but remain speculative and also require confirmation through animal models.

Finally, *Flintibacter*, enriched in VPA users, produces butyrate from glutamine and glutamate and has been linked to seizure suppression in ketogenic diet models.^58^ ^,^ ^59^ Its abundance may reflect microbiota adaptation to ASM exposure and contribute to therapeutic efficacy.

Overall, our results indicate that differences in gut microbiota are detectable at the group level but should not be interpreted as individual-level biomarkers or used for clinical decision-making. Given the exploratory design, limited sample size, and lack of functional validation, these findings require confirmation in larger, independent cohorts and integration with longitudinal and functional studies. Future research should aim to validate these associations and determine whether microbiome-related pathways could be targeted to enhance treatment response as adjunctive therapeutic strategies.

## 4. Conclusion

Our study explored the fecal microbiome across different epilepsy subgroups, focusing on microbial diversity, taxonomic associations, and microbial functional potential in relation to epilepsy phenotypes, ASM use, and treatment responsiveness.

Specific bacterial genera, including *Bacteroides*, *Agathobaculum*, *Bilophila*, and *Coprobacte*r, were associated with treatment-responsive phenotypes. These genera contribute to key processes, including bile acid metabolism, SCFA production, and energy regulation, which may enhance ASM bioavailability and modulate neuroinflammatory and excitatory-inhibitory balance. Moreover, certain taxa, such as *Bacteroides fragilis,* can contribute to neuroprotection by producing metabolites such as GABA, which modulates seizure activity.

Pathways such as inositol synthesis, GABA synthesis III (shunt pathway), SCFA production, and tryptophan degradation via the kynurenine pathway were enriched in treatment-responsive patients, reflecting their roles in neurotransmitter modulation, mitochondrial function, and excitotoxicity prevention. In particular, the contribution of the inositol synthesis pathway to calcium signaling and synaptic vesicle trafficking suggests its importance in maintaining neuronal homeostasis and enhancing ASM efficacy.

Dietary components such as fiber and vitamin B2 (riboflavin) are strongly correlated with beneficial microbial activity and metabolic enhancement. Fiber intake promoted SCFA production and reduced pro-inflammatory taxa, whereas riboflavin was associated with increased abundance of the *Schaalia* genus, potentially contributing to nitric oxide production and mitochondrial health.

These findings advocate for further exploration of microbiota-based interventions, such as prebiotics, probiotics, and dietary modifications, to optimize ASMs efficacy and address treatment resistance. Additionally, microbial and metabolic profiling may help stratify patients based on treatment responsiveness and optimize personalized care for epilepsy.

## 5. Limitations

This study has several limitations. The exploratory design and relatively small, heterogeneous sample limit statistical power and reproducibility. Microbiome profiling based on 16S rRNA sequencing reflects inferred functional potential rather than direct metabolic activity. In addition, potential key confounders such as antiseizure medications, diet, and disease severity may have influenced the results, and the cross-sectional design precludes assessment of temporal relationships and causality. Therefore, these findings are hypothesis-generating and require validation in larger, independent cohorts using longitudinal and functional approaches.

## 6. Materials and methods

Participants were recruited between January 2019 and October 2021. Individuals diagnosed with MTLE, GGE, and AE who were patients at the Neurology outpatient clinic of the Hospital das Clínicas of the University of Campinas (HC-UNICAMP) participated in this study.

### 6.1. Ethical aspects

The Research Ethics Committee of the School of Medical Sciences of UNICAMP (No. 5572034) approved this project as part of the umbrella project: Studies of Molecular Genetics in Neuropsychiatric Diseases Phase I. In addition, the project was registered in the National System of Management of Genetic Heritage and Associated Traditional Knowledge (SisGen) (No. A06FF8A). The patients who participated in the study and their family members were informed about the project and signed written informed consent forms.

### 6.2. Identification and patient selection

Experienced neurologists from the Adult Epilepsy Outpatient Clinic at HC-UNICAMP carried out the clinical evaluation. The definition of epilepsy was based on the recommendations of the ILAE Task Force (2014): "brain disease characterized by any of the following conditions: (1) at least two unprovoked (or reflex) epileptic seizures occurring in an interval greater than 24 hours; (2) an unprovoked (or reflex) epileptic seizure and the probability of occurrence of epileptic seizures similar to the general recurrence risk (of at least 60%) after two unprovoked epileptic seizures, occurring in the next 10 y; (3) diagnosis of an epileptic syndrome"^1^(1).

A meticulous analysis of the patients' semiology was carried out in order to objectify and characterize them into four groups: i) Mesial Temporal Lobe Epilepsy Group (MTLE): patients who presented semiology of seizures with temporal onset, and signs of hippocampal sclerosis on magnetic resonance imaging (MRI), taking into account the presence of volumetric reduction in T1 sequences and/or hypersignal in T2/FLAIR and EEG with compatible activity^60^(13); ii) Generalized Genetic Epilepsy Group (GGE): patients represented by four well-established epileptic syndromes: Childhood Absence Epilepsy, Juvenile Absence Epilepsy, Juvenile Myoclonic Epilepsy, and Epilepsy with Only Tonic-Clonic Seizures^6^ ^,^ ^61^ ^,^ ^62^(6); iii) Autoimmune Encephalitis Group (AE): patients with acute symptomatic seizures secondary to AE or in cases of patients with epilepsy associated with autoimmunity^63^(23), and iv) Control Group (GC): individuals without known neurological diseases, composed mostly of non-related family members of the patients.

### 6.3. Clinical data

Detailed clinical data were obtained from the medical records. It included the age of onset of the disease, seizure frequency, antiseizure medication use, side effects, personal and family pathological history, comorbidities, othermedications, imaging data (MRI, positron emission tomography - PET), electroencephalographic data (EEG), and laboratory results. Patient data were used confidentially.

The eligibility criteria were as follows: i) age between 18 and 70 y; ii) patients classified within the categories MTLE, GGE, and AE; iii) regular follow-up at the HC-UNICAMP epilepsy outpatient clinic; and iv) BMI between 18 and 35. The exclusion criteria were as follows: i) personal history of inflammatory bowel disease or malabsorption syndrome; ii) high dosage of corticosteroids, immunosuppressive agents, and antibiotics (<2 months before collection); iii) consumption of high amounts of probiotics; and iv) gastrointestinal surgery.

The patients were matched by sex and age with healthy individuals belonging to the same family (preferably a spouse), considering that sociodemographic characteristics and eating habits were similar.

### 6.4. Interview, collection, and application of questionnaires

After reading and explanation, written informed consent was obtained from all participants who agreed to donate samples for this study. Subsequently, a customized collection kit was delivered to each participant, which consisted of i) a sterile plastic bag (Coloff®) for isolating the sample in the toilet, ii) a collection tube with a spatula and microbial DNA stabilizer (OMNIgene-GUT OM-200®) that allowed the sample to be preserved for up to 30 days at room temperature, and iii) an instruction manual. The participants watched a simulated demonstration in the doctor's office to ensure that the collection was carried out correctly. The next step involved the administration of two questionnaires. The first, the 24-hour Food Recall, was chosen as a tool to evaluate, define, and quantify all foods and drinks ingested during the period before stool collection. It considers the preparation method, weight, and size of the portions in grams, milliliters, or household measures. Based on the information related to the types and quantities of food, a report was generated detailing the total number of kilocalories, macronutrients (proteins, lipids, and carbohydrates), vitamins, minerals, fiber intake (used to estimate the levels of SCFAs), and trace elements ingested. Nutritional software (WebDiet version 3.0) was used to perform the calculations automatically. Subsequently, a general questionnaire on anthropometric data*, use of medications and/or probiotics, and other aspects considered relevant to the study was applied. The BMI classification of the participants was carried out according to the values recommended by the World Health Organization as follows: i) normal weight: 18.5–24.9; ii) overweight: 25–29.9; iii) obesity type I: 30–34.9.

Finally, all participants were instructed to classify the format of their feces according to the Bristol stool chart. The scale is composed of seven categories according to the format and consistency of feces and aims to verify the intestinal health of individuals. The categories were grouped as follows: i) feces with a tendency to constipation (types 1 and 2), ii) normal feces (types 3 and 4), and iii) feces with a tendency to diarrhea (types 5, 6, and 7).

### 6.5. Sample processing

Once collected, the samples were transported in a thermal box to the Molecular Genetics Laboratory of the Department of Medical Genetics and Genomic Medicine at the School of Medical Sciences - UNICAMP, where they were immediately homogenized, aliquoted, and stored in a biofreezer at −80 °C.

### 6.6. Extraction of microbial genomic DNA

Microbial DNA was extracted using the QIAamp® POWERFECAL® PRO DNA KIT (Qiagen, Hilden, Germany) (80) and quantified using Epoch® (Agilent BioTek Instruments Inc.). The purity and quality of the DNA were measured using spectrophotometry, and the absorbance values of 260/280 ranged between 1.8 and 2.0. The integrity of the DNA was visualized after 1.2% agarose gel electrophoresis.

### 6.7. Metagenomic sequencing

A 16S marker gene approach was carried out to characterize the intestinal microbiota of the individuals included in the study. Genomic libraries were constructed according to the manufacturer's recommendations (Preparing 16S Ribosomal RNA Gene Amplicons for the Illumina MiSeq System, © 2013 Illumina, Inc.).^64^ Selected primers (forward 5′-CCTACGGGNGGCWGCAG-3′ and reverse 5′-GACTACHVGGGTATCTAATCC-3′) were chosen to amplify the target hypervariable region V3-V4 of prokaryotes. Sequencing was performed on an Illumina MiSeq 2500 platform using a v3 flowcell of 600 cycles (MiSeq® Reagent Kit v3, Illumina Inc, San Diego, CA, USA).

### 6.8. Sequencing data processing

The obtained sequences were demultiplexed and contained in the FASTQ files. Quality control of FASTQ sequencing files was performed using FastQC and MultiQC.^65^ Using packages in *R,* ^66^ raw sequence reads were processed by dereplicating and denoising to combine identical reads into amplicon sequence variants (ASVs) and constructing consensus quality profiles for each set of combined sequences using DADA2 (version 3.11).^67^ After removing chimeras, a consensus paired-end reads file was generated for feature construction and downstream analysis. Taxonomic binning of the classified sequences was conducted using a local copy of the Ribosomal Database Project (RDP) Classifier (Train Set 19).^68^ Normalized data were derived from the relative abundance of taxa in each sample. Consequently, a feature table comprising 5368 unique ASVs with an average read length of 410 nucleotides across 96 samples was constructed. Following pre-processing, the minimum number of reads per sample was 27292, with an average of 66767 reads per sample.

### 6.9. Statistical analysis

Management and analyses of patient metadata were performed using the SPSS program (IBM Corp., Released 2013. IBM SPSS Statistics for Windows, Version 29.0.2.0 Armonk, NY: IBM Corp.) and *R* packages (V.2.15.3). Data were analyzed for Gaussian distribution using the Shapiro-Wilk test. For demographic characteristics, the chi-square test and Student's t-test were used for dichotomous and continuous parametric variables, respectively. Moreover, Fisher’s exact test was utilized to compare categorical variables in small-sized groups and the Kruskal–Wallis test to assess differences between more than two independent groups.

Sequencing data was analyzed using bioinformatics and statistical analysis packages in R, including vegan (version 2.6.4),^69^ dada2 (version 3.18),^67^ phyloseq (version 1.46.0),^70^ ggplot2 (version 3.4.4),^71^ and CoDaSeq (version 0.99.7).^72^ ^,^ ^73^ Simpson's index was calculated to examine the alpha diversity of the microbial communities (using the estimate_richness function from the phyloseq package). The Wilcoxon rank-sum test was used to determine the differences among groups.^74^ Taxa were aggregated at the genus level with species-level assignments, where feasible. To standardize the differences in sequencing depth per sample, the data were transformed to relative abundances scaled to 100%. Variance filtering was applied using the genefilter function (version 1.84.0), which excluded taxa with the lowest 40% variance values. Abundance matrices underwent centered log-ratio (clr) transformation (using codaSeq.clr in the CoDaSeq package), with zeros imputed based on the minimum proportional abundance detected for each taxon. Community variation was visualized using multidimensional scaling (MDS) based on Aitchison distances at the genus level of classification. The contribution of metadata variables to microbiome community variation was assessed using the capscale function from the vegan package.^69^

The ASV table was filtered to retain taxa observed in at least 15% of the participants. The relationship between taxonomic abundance and metadata variables was assessed using linear regression models implemented with the fw_glm function from the Tjazi package.^75^ Adjustments were made for covariates such as age, sex, BMI, and Bristol stool scale (BSS) scores. Statistical significance was defined as a false discovery rate (FDR)-corrected *q-value* ≤ 0.1.

Metagenomes of the intestinal microbiome were imputed from 16S rRNA sequences using the Phylogenetic Investigation of Communities by Reconstruction of Unobserved States (PICRUSt) software.^76^ This method predicts the abundance of orthologous gene families from phylogenetic information with an estimated accuracy of 0.8. A table of representative OTUs was used for metagenomic imputation. The resulting OTU table was then normalized by the number of copies of the 16S rRNA gene. The gene content was predicted for each individual sample. Subsequently, the predicted functional (metabolic) composition profiles were compared with the pathways available in the Kyoto Encyclopedia of Genes and Genomes (KEEG) database.^77^ PICRUSt (Phylogenetic Investigation of Communities by Reconstruction of Unobserved States) analysis was conducted to predict the functional potential of microbial communities, specifically focusing on GBMs, providing insights into the metabolic capabilities that could influence the central nervous system and the contributions of different taxa within the microbial community.^78^

## Supplementary Material

Supplementary Material Supplemental _material.pdf

## Data Availability

The cohort metadata presented in this study are available in the Supplementary Material. See also: https://drive.google.com/drive/folders/1-uj4ZAkeVsv2CBsBjR_3wtpPSVh8iDPM?usp=drive_link.

## Data deposition

16s sequencing data generated in this study are available in the NCBI repository under the accession number PRJNA1190103. Temporary Submission ID: SUB14876304 Release date: 2026-07-01.
