# Supplementary material for: Intestinal microbial community assessment in patients with different forms of epilepsy and autoimmune encephalitis: An exploratory study
Source: Gut Microbes Rep. 2026 May 28;3(1):2679348. doi: 10.1080/29933935.2026.2679348 (PMC13224764; doi:10.1080/29933935.2026.2679348)
Supplement: Supplementary Material — Supplemental _material.pdf [file KGMR_A_2679348_SM9117.pdf]

Table 1. Sociodemographic, clinical, and nutritional characteristics of patients with epilepsy (MTLE + GGE + AE) and the control group.

		Group		
		**Control** (N= 37, 38.5%)	**Patient with epilepsy** (N=59, 61.5%)	***p*-value**
**Sex (N, %)**	Male	16 (16.7%)	20 (20.8%)	0.357[a]
	Female	21 (21.9%)	39 (40.6%)	
**BMI (N, %)**	Normal	19 (19.8%)	29 (30.2%)	0.577 [a]
	Overweight	12 (12.5%)	24 (25.0%)	
	Obesity 1	6 (6.3%)	6 (6.3%)	
**Obese (N, %)**	No	31 (32.3%)	53 (55.2%)	0.383 [a]
	Yes	6 (6.3%)	6 (6.3%)	
**Bristol_Classification (N, %)**	Constipation	8 (8.3%)	15 (15.6%)	0.832 [a]
	Normal	27 (28.1%)	42 (43.8%)	
	Diarrhea	2 (2.1%)	2 (2.1%)	
**Age (mean, ±SD)**		42.2±12.3	45.6±16.7	0.283 [b]
**BMI**		25.6±3.5	25.1±3.7	0.578 [b]
**Proteins (g)**		85.3±31.8	74.5±31.01	0.102 [b]
**Lipids (g)**		42.7±19.8	38.6±16.7	0.279 [b]
**Carbohydrates (g)**		167.4±51.4	162.2±43.1	0.596 [b]
**Daily_calories (kcal)**		1355.5±407.1	1255.8±310.4	0.179 [b]
**MUFAs (g)**		12.8±5.6	11.3±6.08	0.232 [b]
**PUFAs (g)**		7.0±3.7	6.6±4.02	0.581 [b]
**SFA (g)**		13.9±6.0	12.9±6.0	0.445 [b]
**Trans_FA (g)**		1.8±4.6	0.7±0.5	0.084 [b]
**Colesterol (mg)**		297.1±215.5	245.0±159.5	0.178 [b]
**Fibers (g)**		21.7±8.4	20.1±9.4	0.387 [b]
**Calcium (mg)**		531.1±297.8	448.5±231.5	0.132 [b]
**Magnesium (mg)**		253.9±90.1	219.8±76.8	0.051 [b]
**Phosphorus (mg)**		1025.1±333.7	893.0±345.7	0.068 [b]
**Iron (mg)**		9.6±7.9	8.0±4.6	0.230 [b]
**Sodium (mg)**		1303.9±896.8	1161.7±771.1	0.411 [b]
**Potassium (mg)***		2559.6±915.9	2142.4±647.8	0.010 [b]
**Copper (mg)**		1.3±1.1	1.4±2.1	0.732 [b]
**Zinc (mg)***		9.4±4.3	7.7±3.8	0.042 [b]
**Selenium (mcg)**		92.6±161.6	64.4±165.8	0.415 [b]
**Vitamin_A (mcg)**		589.6±465.7	692.6±2121.2	0.772 [b]
**Vitamin_B1 (mg)***		0.93±0.64	0.68±0.28	0.012 [b]
**Vitamin_B2 (mg)**		1.21±0.60	1.04±0.64	0.201 [b]
**Vitamin_B3 (mg)**		21.3±16.5	19.1±18.4	0.553 [b]
**Vitamin_B6 (mg)***		0.71±0.49	0.47±0.35	0.007 [b]
**Vitamin_B9 (mcg)**		318.0±244.1	312.3±131.5	0.884 [b]
**Vitamin_B12 (mcg)**		4.21±6.6	4.51±11.2	0.884 [b]

**Vitamin_C (mg)**		111.1±96.6	85.2±96.9	0.204 [b]
**Vitamin_D (mcg)**		2.52±2.42	1.92±1.99	0.194 [b]
**Vitamin_E (mg)***		7.09±6.79	4.44±2.30	0.007 [b]

AE: autoimmune encephalitis. BMI: body mass index. GGE: genetic generalized epilepsy. g: grams. kcal: kilocalorie. mcg: micrograms. mg: milligrams. MTLE: mesial temporal lobe epilepsy. SD: standard deviation.

[a] Chi-square test. [b] Student's t-test.

Table 2. Number of antiseizure medications, treatment responsiveness, and location of hippocampal atrophy according to epilepsy group.

		Classification			*p*-value
		**MTLE**	**GGE**	**AE**	
**N° of ASMs**	**Negative**	1 (1.7%)	0 (0.0%)	1 (1.7%)	0.045
	**Monotherapy**	5 (8.5%)	7 (11.9%)	2 (3.4%)	
	**2 ASMs**	8 (13.6%)	1 (1.7%)	3 (5.1%)	
	**> 2 ASMs**	24 (40.7%)	3 (5.1%)	4 (6.8%)	
**CBZ**	**No**	25 (42.4%)	11 (18.6%)	6 (10.2%)	0.014
	**Yes**	13 (22.0%)	0 (0.0%)	4 (6.8%)	
**CLB**	**No**	7 (11.9%)	9 (15.3%)	4 (6.8%)	< 0.001
	**Yes**	31 (52.5%)	2 (3.4%)	6 (10.2%)	
**LEV**	**No**	28 (47.5%)	11 (18.6%)	9 (15.3%)	0.04
	**Yes**	10 (16.9%)	0 (0.0%)	1 (1.7%)	
**LTG**	**No**	18 (30.5%)	5 (8.5%)	9 (15.3%)	0.028
	**Yes**	20 (33.9%)	6 (10.2%)	1 (1.7%)	
**TPM**	**No**	28 (47.5%)	9 (15.3%)	9 (15.3%)	0.474
	**Yes**	10 (16.9%)	2 (3.4%)	1 (1.7%)	
**VPA**	**No**	28 (47.5%)	4 (6.8%)	8 (13.6%)	0.052
	**Yes**	10 (16.9%)	7 (11.9%)	2 (3.4%)	
**Responsiveness**	**Responsive**	3 (5.1%)	8 (13.6%)	5 (8.5%)	< 0.001
	**Refractory**	35 (59.3%)	3 (5.1%)	5 (8.5%)	
**MRI with Hippocampal atrophy**	**Normal**	1 (1.7%)	11 (18.6%)	3 (5.1%)	< 0.001
	**Right**	13 (22.0%)	0 (0.0%)	1 (1.7%)	
	**Left**	19% (32.2%)	0 (0.0%)	4 (6.8%)	
	**Bilateral**	5 (8.5%)	0 (0.0%)	2 (3.4%)	

AE: autoimmune encephalitis. ASMs: antiseizure medications. BMI: body mass index. CBZ: carbamazepine. CLB: clobazam. GGE: genetic generalized epilepsy. g: grams. kcal: kilocalorie. LEV: levetiracetam. LTG: lamotrigine. mcg: micrograms. mg: milligrams. MTLE: mesial temporal lobe epilepsy. MRI: magnetic resonance imaging SD: standard deviation. TPM: topiramate. VPA: valproic acid.
